# Evaluation of Montanide™ ISA 71 VG Adjuvant during Profilin Vaccination against Experimental Coccidiosis

**DOI:** 10.1371/journal.pone.0059786

**Published:** 2013-04-08

**Authors:** Seung I. Jang, Duk Kyung Kim, Hyun S. Lillehoj, Sung Hyen Lee, Kyung Woo Lee, François Bertrand, Laurent Dupuis, Sébastien Deville, Juliette Ben Arous, Erik P. Lillehoj

**Affiliations:** 1 Animal Parasitic Diseases Laboratory, Animal and Natural Resources Institute, Agricultural Research Service-U.S. Department of Agriculture, Beltsville, Maryland, United States of America; 2 SEPPIC, 22 Terrasse Bellini, 92800 Puteaux, France; 3 Department of Pediatrics, University of Maryland School of Medicine, Baltimore, Maryland, United States of America; University of Pittsburgh, United States of America

## Abstract

Chickens were immunized subcutaneously with an *Eimeria* recombinant profilin protein plus Montanide™ ISA 70 VG (ISA 70) or Montanide™ ISA 71 VG (ISA 71) water-in-oil adjuvants, or with profilin alone, and comparative RNA microarray hybridizations were performed to ascertain global transcriptome changes induced by profilin/ISA 70 *vs*. profilin alone and by profilin/ISA 71 *vs*. profilin alone. While immunization with profilin/ISA 70 *vs*. profilin alone altered the levels of more total transcripts compared with profilin/ISA 71 *vs*. profilin alone (509 *vs*. 296), the latter was associated with a greater number of unique biological functions, and a larger number of genes within these functions, compared with the former. Further, canonical pathway analysis identified 10 pathways that were associated with genes encoding the altered transcripts in animals immunized with profilin/ISA 71 *vs*. profilin alone, compared with only 2 pathways in profilin/ISA 70 *vs*. profilin alone. Therefore, ISA 71 was selected as a candidate adjuvant in conjunction with profilin vaccination for *in vivo* disease protection studies. Vaccination with profilin/ISA 71 was associated with greater body weight gain following *E. acervulina* infection, and decreased parasite fecal shedding after *E. maxima* infection, compared with profilin alone. Anti-profilin antibody levels were higher in sera of *E. maxima*- and *E. tenella*-infected chickens vaccinated with profilin/ISA 71 compared with profilin alone. Finally, the levels of transcripts encoding interferon-γ, interleukin (IL)-2, IL-10, and IL-17A were increased in intestinal lymphocytes from *E. acervulina*-, *E. maxima*-, and/or *E. tenella*-infected chickens vaccinated with profilin/ISA 71 compared with profilin alone. None of these effects were seen in chickens injected with ISA 71 alone indicating that the adjuvant was not conferring non-specific immune stimulation. These results suggest that profilin plus ISA 71 augments protective immunity against selective *Eimeria* species in chickens.

## Introduction

Avian coccidiosis is one of the most costly infectious diseases affecting the commercial poultry industry [1]. Coccidia that infect chickens include *Eimeria acervulina*, *E. tenella*, *E. maxima*, *E. brunetti*, *E. necatrix*, *E. praecox*, and *E. mitis*. These apicomplexan protists invade cells of the intestinal epithelium, evoking necrotic tissue destruction and resulting in reduced body weight gain in broilers, decreased egg production in layers, and fecal shedding of viable parasites that re-infect susceptible animals upon ingestion. Over the preceding 40 years, avian coccidiosis has been mainly controlled by prophylactic chemotherapeutic drugs. More recently, the use of coccidia vaccines has reduced the need for in-feed medication [2]. Because host immunity to *Eimeria* infection is species-specific, currently available live, attenuated vaccines consist of mixtures of four or more *Eimeria* species. The basis of using live coccidia vaccines involves a continuous excretion/re-ingestion cycle of an initial low dose of parasites, which progressively induces protective flock immunity. However, live vaccines often lead to an early reduction in weight gain and may not be effective against regional antigenic variants absent from the formulation. Therefore, novel approaches are needed to more effectively control coccidiosis in commercial poultry flocks.

Vaccine delivery in conjunction with an adjuvant offers one means to increase potency. By definition, an adjuvant is an agent that stimulates the immune system and increases the host response to an antigen without itself conferring a specific antigenic effect [3]. Some adjuvants act by sequestering antigens in physically restricted areas, termed depots, to provide an extended time period of antigenic stimulation. This depot effect is essential for the efficacy of the majority of human and veterinary vaccine adjuvants, particularly with vaccines consisting of pathogen subunits (proteins, nucleic acids, and carbohydrates). Currently, aluminum hydroxide (alum) is only adjuvant approved for human use in the U.S. and Canada. Other adjuvants have been licensed for human use in Europe, including the water-in-oil (W/O) emulsions, MF59 and AS03, and the TLR4 agonist, monophosphoryl lipid A in alum [4]. A larger list of adjuvants is available for veterinary use [5].

The Montanide ISA series of adjuvants include the W/O emulsions, Montanide™ ISA 70 VG (ISA 70) and Montanide™ ISA 71 VG (ISA 71). Both formulations are mineral oil-based solutions incorporating a highly refined mannitol/oleic acid emulsifier [6]. ISA 71 is similar to ISA 70 except that it contains an improved mineral oil enabling the preferential stimulation of Th1-type cell-mediated immunity. ISA 70 and ISA 71 have been successfully applied to enhance immune response against pathogens of poultry, cattle, and small ruminants [7]. Our previous studies demonstrated that either ISA 70 or ISA 71 in conjunction with the *Eimeria* recombinant protein, profilin, was associated with enhanced protective immunity against experimental avian coccidiosis, as measured by increased body weight gain and/or decreased fecal oocyst shedding compared with profilin alone [8], [9], [10]. However, the specific effects were dependent upon the particular adjuvant used, the species of infecting *Eimeria*, and the parameter of infection measured. Profilin/ISA 70 and profilin/ISA 71 increased post-infection weight gains, but only following *E. acervulina* infection, whereas profilin/ISA 71, but not profilin/ISA 70, decreased parasite shedding following infection with *E. acervulina* or *E. tenella*. In addition, we have demonstrated the utility of comparative microarray hybridization for identifying global transcriptional responses to various vaccination strategies that correlate with protection against experimental *Eimeria* infection [11], [12], [13]. Therefore, the current study was undertaken to compare the dynamics of lymphocyte transcriptome responses in chickens immunized with profilin/ISA 70 *vs*. profilin alone, or with profilin/ISA 71 *vs*. profilin alone, and to use this information to identify the better adjuvant with the potential for stimulating protective immunity against experimental avian coccidiosis.

## Materials and Methods

### Recombinant Profilin Protein and Adjuvants

Recombinant profilin, originally derived from *E. acervulina*, was expressed in *Escherichia coli* with a maltose binding protein epitope tag and purified by amylase affinity chromatography as described [8]. Purified profilin was emulsified with ISA 70 or ISA 71 at a 30∶70 ratio (w:w, profilin:adjuvant) as recommended by the manufacturer (Seppic, Puteaux, France).

### Chickens and Profilin Immunization

One-day-old Ross broiler chickens (Longenecker’s Hatchery, Elizabethtown, PA) were housed in Petersime starter brooder units and provided with feed and water *ad libitum*. At 7 days post-hatch, chickens were subcutaneously immunized with 50 μg of profilin emulsified in ISA 70 or ISA 71. Control chickens were immunized with PBS plus adjuvant, or with profilin in the absence of adjuvant. At 7 days post-primary immunization, the chickens were transferred to hanging cages (2 birds per cage) and given secondary subcutaneous booster injections identical with the primary immunization. All experiments were approved by the Beltsville Agricultural Research Center Small Animal Care and Use Committee.

### Total RNA Preparation and Labeling

At 7 days post-secondary immunization, birds were sacrificed, single spleen cell suspensions were prepared, and lymphocytes were isolated by Percoll density gradient centrifugation as described [14]. Total RNA was isolated using Trizol (Invitrogen, Carlsbad, CA) and pooled into 2 equal aliquots (3 birds/sample). RNAs were amplified using the Two-Color Quick Amp Labeling Kit (Agilent Technologies, Santa Clara, CA) with cyanine 3 (Cy3)- or Cy5-labeled CTP. Labeled RNAs were purified using the RNeasy Mini Kit (Qiagen, Valencia, CA) and quantified with a Nanodrop ND-1000 UV-VIS spectrophotometer (Thermo Fisher Scientific, Waltham, MA).

### Microarray Experimental Design

A standard reference design with hybridization of Cy3- and Cy5-labeled RNAs [15] was used to compare mRNA transcript levels in chickens immunized with profilin/ISA 70 *vs*. profilin alone and profilin/ISA 71 *vs*. profilin alone. Two biological replicates were conducted in each comparison with substitution of Cy3- and Cy5-labeled RNAs to prevent data distortion from sample labeling as previously described [12]. Labeled RNAs were hybridized to a Chicken Gene Expression Microarray (Agilent Technologies, Santa Clara, CA) containing 43,803 elements using the Gene Expression Hybridization Kit (Agilent Technologies) with constant mixing at 10 rpm for 17 hr at 65°C. After washing, microarray images were scanned, and data extraction and analysis were performed using Feature Extraction software version 10.7.3.1 (Agilent Technologies).

### Microarray Data Analysis

GeneSpring GX10 software (Silicon Genetics, Redwood, CA) was used to qualify and normalize hybridization images, and to perform fold-change analyses as described [11], [12]. Median signal intensities were qualified by subtracting the median local background and normalized by locally-weighted scatterplot smoothing (LOWESS). Flag information was applied to strain the spots with 100% valid values from each group and one-way analysis of variance (ANOVA) was performed to analyze the significance of differences between treatment groups. To generate signal ratios, signal channel values from the profilin/ISA 70 and profilin/ISA 71 groups were divided by the channel values from the profilin-only group. Modulated mRNA transcripts, defined as those with a cutoff of *P*<0.0005, were applied by asymptotic T-test analysis. The significantly differentially expressed transcripts were filtered using the Volcano Plot method [16] built by comparison among the various immunization groups. All microarray information and data was deposited online into the Gene Expression Omnibus (GEO) server (accession number GES 40743).

### Bioinformatic Analysis

Differentially expressed transcripts were analyzed by Ingenuity Pathway Analysis (IPA) software (Ingenuity Systems, Redwood City, CA). Each identifier was mapped to its corresponding gene object in the Ingenuity Knowledge Base. Both up-regulated and down-regulated identifiers were defined as value parameters for the analysis. These focus genes were overlaid onto a global molecular network developed from information contained in the Ingenuity Knowledge Base. Biological functional analysis was performed and the canonical pathways from the datasets were mapped with the Ingenuity Pathways Knowledge Base. The Fischer’s exact test was used to calculate the probability that each biological function and associated pathways assigned to that dataset was statistically significant.

### Experimental Eimeria Infection

At 7 days post-secondary immunization, chickens were uninfected or were orally infected with 1.0×10^4^ sporulated oocysts of *E. acervulina, E. tenella* or *E. maxima* as described [17]. The coccidia parasites were originally isolated and maintained at the Animal and Natural Resources Institute, U.S. Department of Agriculture (Beltsville, MD). Prior to infection, sporulated oocysts were cleaned by floatation on 2.5% sodium hypochlorite, washed three times with PBS, and enumerated using a hemocytometer.

### Body Weight Gains and Fecal Oocyst Shedding

Body weights of uninfected and *Eimeria*-infected chickens (8/group) were measured at 0 and 10 days post-infection. For determination of fecal oocysts numbers, birds (8/group) were placed on wire oocyst collection cages, fecal samples were collected between 6 and 10 days post-infection, and total oocysts were enumerated using a McMaster counting chamber as described [18].

### Anti-profilin Serum Antibody Levels

At 3 days post-infection, chickens (5/group) were euthanized by cervical dislocation, blood was collected by cardiac puncture, and sera were prepared by low speed centrifugation. Serum antibodies against profilin were measured by enzyme-linked immunosorbent assay (ELISA) as described [10], [18]. Ninety-six-well microtiter plates were coated overnight with 10 μg/well of purified recombinant profilin, washed with PBS containing 0.05% Tween 20, and blocked with PBS containing 1% BSA. Serum samples were added and incubated for 1 hr with continuous shaking, the plates were washed, and bound antibody was detected with peroxidase-conjugated rabbit anti-chicken IgG secondary antibody and peroxidase-substrate (Sigma, St. Louis, MO). Optical density (OD) values at 450 nm were measured with an automated microplate reader (Bio-Rad, Richmond, CA).

### Intestinal Cytokine mRNA Levels

At 3 day post-infection, chickens (3/group) were euthanized by cervical dislocation and the intestinal duodenum (*E. acervulina*-infected), jejunum (*E. tenella*-infected), and cecum (*E. maxima*-infected) were removed. Tissues were incised longitudinally, gently washed with ice-cold Hank’s Balanced Salt Solution (Sigma), and intraepithelial lymphocytes (IELs) were isolated by density gradient centrifugation as described [19], [20]. Total RNA was isolated, 5.0 μg were incubate with 1.0 U of DNase I and 1.0 μl of 10× DNase I reaction buffer (Sigma) for 15 min at room temperature, 1.0 μl of stop solution was added, and the mixture was heated at 70°C for 10 min. RNA was reverse-transcribed using the StrataScript first-strand synthesis system (Stratagene, La Jolla, CA) according to the manufacturer’s recommendations. PCR amplification and detection were carried out using equivalent amounts of total RNA and oligonucleotide primers for IFN-γ, IL-2, IL-10, IL-17A, and the glyceraldehydes-3-phosphate dehydrogenase (GAPDH) internal control (Table 1) with the Mx3000P system and Brilliant SYBR Green QPCR master mix (Stratagene). Standard curves were generated using log_10_ diluted standard RNAs and the levels of individual transcripts were normalized to those of GAPDH by the Q-gene program [21]. To normalize RNA levels between samples within an experiment, the mean threshold cycle (C_t_) values for the amplification products were calculated by pooling values from all samples in that experiment. Each analysis was performed in triplicate.

**Table 1:** Oligonucleotide primers used for quantitative RT-PCR of chicken cytokine transcripts.

RNA Target	Primer Sequences	PCR Product Size (bp)	GenBank Accession No.
IFN- γ			
Forward	5′-AGCTGACGGTGGACCTATTATT-3′	259	Y07922
Reverse	5′-GGCTTTGCGCTGGATTC-3′		
IL-2			
Forward	5′-TCTGGGACCACTGTATGCTCT-3′	256	AF000631
Reverse	5′-ACACCAGTGGGAAACAGTATCA-3′		
IL-10			
Forward	5′-CGGGAGCTGAGGGTGAA-3′	272	AJ621614
Reverse	5′-GTGAAGAAGCGGTGACAGC-3′		
IL-17A			
Forward	5′-CTCCGATCCCTTATTCTCCTC-3′	292	AJ493595
Reverse	5′-AAGCGGTTGTGGTCCTCAT-3′		
GAPDH			
Forward	5′-GGTGGTGCTAAGCGTGTTAT-3′	264	K01458
Reverse	5′-ACCTCTGTCATCTCTCCACA-3′		

### Statistical Analysis

Body weight gains, oocyst shedding, anti-profilin antibody titers, and cytokine levels were expressed as means ± SD. Mean values of different treatment groups were compared using ANOVA or the Duncan’s multiple range test with SPSS 15.0 for Windows (SPSS Inc., Chicago, IL). Differences between means were considered statistically significant at *P*<0.05.

## Results

### Spleen Lymphocyte Transcript Levels Following Immunization with Profilin Plus ISA 70 or Profilin Plus ISA 71

**Figure 1:**
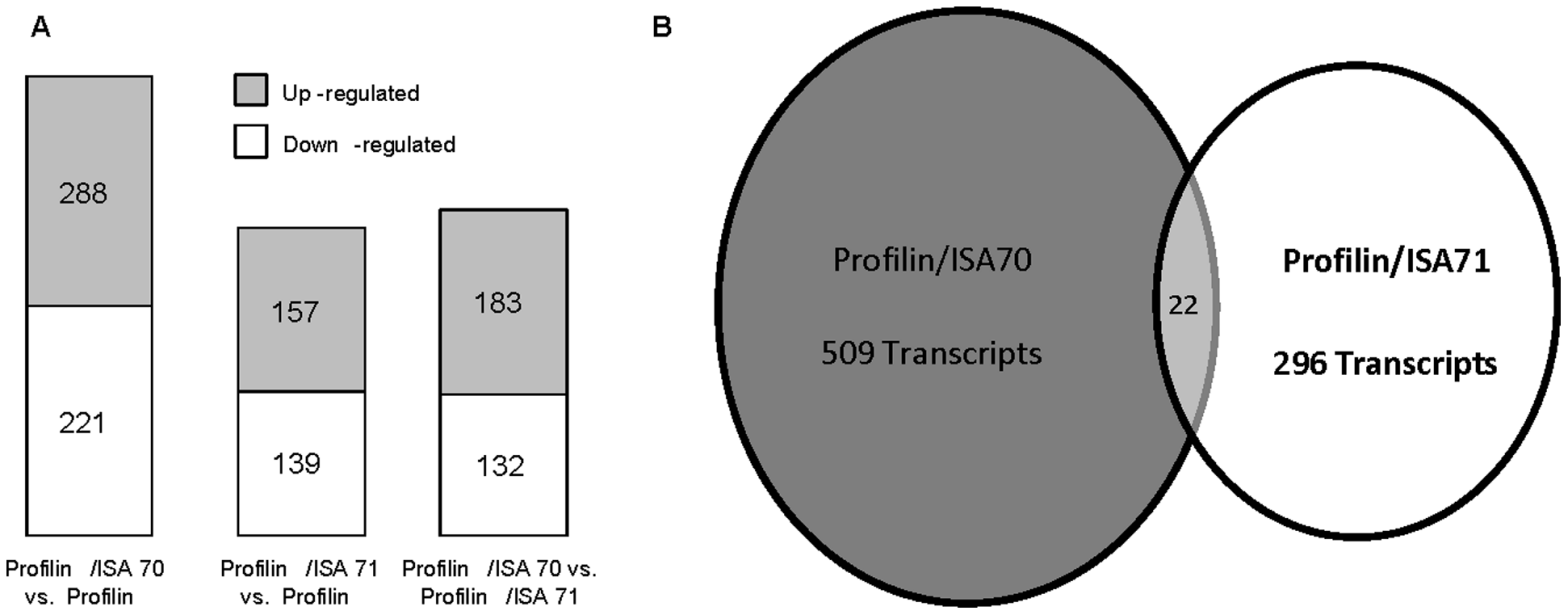
Comparison of the numbers of differentially altered transcript levels in spleen lymphocytes when comparing chickens immunized with profilin plus ISA 70 *vs*. profilin alone, profilin plus ISA 71 *vs*. profilin alone, or profilin plus ISA 70 *vs*. profilin plus ISA 71. (A) The numbers of up-regulated and down-regulated transcripts. (B) The number of identical transcripts.

Microarray hybridizations were performed using the Agilent Technology Chicken Gene Expression Microarray with total RNAs isolated from spleen lymphocytes of chickens immunized with profilin alone, profilin plus ISA 70, or profilin plus ISA 71 to identify global transcriptome changes in the respective treatment groups. A critical *P* value of 0.0005 was employed to compare transcript levels in the profilin/ISA 70 *vs*. profilin alone, the profilin/ISA 71 *vs*. profilin alone, and the profilin/ISA 70 *vs*. profilin/ISA 71 groups. From this dataset, the levels of 509 (288 up-regulated, 221 down-regulated), 296 (157 up-regulated, 139 down-regulated), and 315 (183 up-regulated, 132 down-regulated) mRNAs were identified as differentially altered in the denoted comparisons (Figure 1A). Twenty-two altered transcripts were identical in the profilin/ISA 70 *vs*. profilin alone (11 up-regulated, 11 down-regulated) and the profilin/ISA 71 *vs*. profilin alone (10 up-regulated, 12 down-regulated) groups (Figure 1B). Tables 2 and 3 list the 20 most up-regulated and 20 most down-regulated transcripts for these latter two comparisons in decreasing order of fold-change.

**Table 2:** Differential gene transcript levels comparing profilin plus ISA 70 *vs*. profilin alone.

Symbol	Entrez Gene Name	Fold-Change^1^	Location	Function
Up-regulated				
UPK3BL	Uroplakin 3B-like	6.744	Unknown	Other
C1QTNF3	C1q and tumor necrosis factor related protein 3	5.840	Extracellular Space	Other
APOLD1	Apolipoprotein L domain containing 1	4.682	Unknown	Other
CHRDL1	Chordin-like 1	4.044	Extracellular Space	Other
PIGR	Polymeric immunoglobulin receptor	3.942	Plasma Membrane	Transporter
EPCAM	Epithelial cell adhesion molecule	3.669	Plasma Membrane	Other
SCGN	Secretagogin, EF-hand calcium binding protein	3.645	Cytoplasm	Other
IRX4	Iroquois homeobox 4	3.597	Nucleus	Transcr. regulator
RBM6	RNA binding motif protein 6	2.857	Nucleus	Other
Pdlim3	PDZ and LIM domain 3	2.789	Plasma Membrane	Other
ADPRHL1	ADP-ribosylhydrolase like 1	2.687	Unknown	Enzyme
FMOD	Fibromodulin	2.493	Extracellular Space	Other
LIMA1	LIM domain and actin binding 1	2.368	Cytoplasm	Other
Krt19	Keratin 19	2.356	Cytoplasm	Other
SCRN1	Secernin 1	2.347	Cytoplasm	Other
LMCD1	LIM and cysteine-rich domains 1	2.315	Cytoplasm	Transcr. regulator
FAM40B	Family with sequence similarity 40, member B	2.311	Unknown	Other
ARL8A	ADP-ribosylation factor-like 8A	2.274	Cytoplasm	Enzyme
C11orf96	Chromosome 11 open reading frame 96	2.221	Unknown	Other
STMN2	Stathmin-like 2	2.161	Cytoplasm	Other
Down-regulated				
ART1	ADP-ribosyltransferase 1	−18.34	Plasma Membrane	Enzyme
MMP7	Matrix metallopeptidase 7 (matrilysin, uterine)	−4.322	Extracellular Space	Peptidase
CRTC1	CREB regulated transcription coactivator 1	−3.184	Nucleus	Transcr. regulator
ID2	Inhibitor of DNA binding 2, DN helix-loop-helix protein	−2.479	Nucleus	Transcr. regulator
ALDH9A1	Aldehyde dehydrogenase 9 family, member A1	−2.274	Cytoplasm	Enzyme
TBC1D2B	TBC1 domain family, member 2B	−2.242	Unknown	Other
DEF6	Differentially expressed in FDCP 6 homolog	−2.240	Extracellular Space	Other
FBXO18	F-box protein, helicase, 18	−2.218	Nucleus	Other
MT-CO2	Cytochrome c oxidase subunit II	−2.116	Cytoplasm	Enzyme
TMEM144	Transmembrane protein 144	−1.870	Unknown	Other
SQSTM1	Sequestosome 1	−1.869	Cytoplasm	Transcr. regulator
VDR	Vitamin D (1,25- dihydroxyvitamin D3) receptor	−1.865	Nucleus	Nuclear receptor
IL8	Interleukin 8	−1.836	Extracellular Space	Cytokine
C19orf28	Chromosome 19 open reading frame 28	−1.796	Unknown	Other
ATP5B	ATP synthase, H^+^ transporting, mitochondrial, β	−1.782	Cytoplasm	Transporter
RRBP1	Ribosome binding protein 1 homolog 180 kDa (dog)	−1.733	Cytoplasm	Transporter
PARP4	Poly (ADP-ribose) polymerase family, member 4	−1.702	Nucleus	Enzyme
C9orf102	Chromosome 9 open reading frame 102	−1.701	Unknown	Enzyme
HYAL2	Hyaluronoglucosaminidase 2	−1.696	Cytoplasm	Enzyme
CNOT1	CCR4-NOT transcription complex, subunit 1	−1.689	Cytoplasm	Other

1 Values are the log_2_ of the ratio of gene transcript levels (profilin/ISA 70 ÷ profilin alone).

**Table 3:** Differential gene transcript levels comparing profilin plus ISA 71 *vs*. profilin alone.

Symbol	Entrez Gene Name	Fold-Change^1^	Location	Function
Up-regulated				
SST	Somatostatin	6.132	Extracellular Space	Other
GCG	Glucagon	4.113	Extracellular Space	Other
CHST12	Carbohydrate (chondroitin 4) sulfotransferase 12	2.643	Cytoplasm	Enzyme
LSP1	Lymphocyte-specific protein 1	2.435	Cytoplasm	Other
F7	Coagulation factor VII	2.431	Plasma Membrane	Peptidase
TLX1	T-cell leukemia homeobox 1	2.136	Nucleus	Transcr. regulator
GP9	Glycoprotein IX (platelet)	2.112	Plasma Membrane	Other
FAM40B	Family with sequence similarity 40, member B	2.047	Unknown	Other
FDFT1	Farnesyl-diphosphate farnesyltransferase 1	2.031	Cytoplasm	Enzyme
COL17A1	Collagen, type XVII, α 1	1.947	Plasma Membrane	Other
ARL8A	ADP-ribosylation factor-like 8A	1.914	Cytoplasm	Enzyme
TERT	Telomerase reverse transcriptase	1.893	Nucleus	Enzyme
PRKCH	Protein kinase C, β	1.836	Cytoplasm	Kinase
TTC9	Tetratricopeptide repeat domain 9	1.813	Unknown	Other
HSD11B1	Hydroxysteroid (11-β) dehydrogenase 1	1.805	Cytoplasm	Enzyme
EPSTI1	Epithelial stromal interaction 1 (breast)	1.792	Unknown	Other
IRAK1BP1	IL-1 receptor-associated kinase 1 binding protein 1	1.758	Unknown	Other
TUBB1	Tubulin, β 1	1.731	Cytoplasm	Other
CCDC81	Coiled-coil domain containing 81	1.728	Unknown	Other
P4HA3	Polyl 4-hydroxylase, alpha polypeptide III	1.691	Unknown	Enzyme
Down-regulated				
RAB14	RAB14, member RAS oncogene family	−5.333	Cytoplasm	Enzyme
SFTPA1	Surfactant protein A1	−3.568	Extracellular Space	Transporter
USP32	Ubiquitin specific peptidase 32	−2.270	Cytoplasm	Enzyme
PDE5A	Posphodiesterase 5A, cGMP-specific	−2.229	Cytoplasm	Enzyme
AHR	Aryl hydrocarbon receptor	−2.044	Nucleus	Nuclear receptor
GOLGB1	Golgin B1	−1.959	Cytoplasm	Other
ABI3BP	ABI family, member 3 (NESH) binding protein	−1.953	Extracellular Space	Other
XPO1	Exportin 1 (CRM1 homolog, yeast)	−1.774	Nucleus	Transporter
ATP6V0D2	ATPase, H^+^ transporting, lysosomal 38 kDa, subunit d2	−1.766	Cytoplasm	Transporter
VWF	Von Willebrand factor	−1.757	Extracellular Space	Other
PHACTR1	Phosphatase and actin regulator 1	−1.724	Cytoplasm	Other
NUP153	Nucleoporin 153 kDa	−1.720	Nucleus	Transporter
FAM91A1	Family with sequence similarity 91, member A1	−1.647	Unknown	Other
ARID4A	AT rich interactive domain 4A (RBP1-like)	−1.579	Nucleus	Transcr. regulator
PROC	Protein C (inactivator of coagulation factors Va, VIIIa)	−1.575	Extracellular Space	Peptidase
PIAS1	Protein inhibitor of activated STAT, 1	−1.525	Nucleus	Transcr. regulator
NUP155	Nucleoporin 155 kDa	−1.512	Nucleus	Transporter
MLLT4	Myeloid/lymphoid or mixed-lineage leukemia	−1.506	Nucleus	Other
NAPG	N-ethylmaleimide-sensitive factor attachment protein,γ	−1.506	Cytoplasm	Transporter
AMN1	Antagonist of mitotic exit network 1 homolog	−1.454	Plasma Membrane	Other

1 Values are the log_2_ of the ratio of gene transcript levels (profilin/ISA 71 ÷ profilin alone).

### Biological Function and Pathway Analysis of Differentially Regulated Splenocyte Transcripts

**Figure 2:**
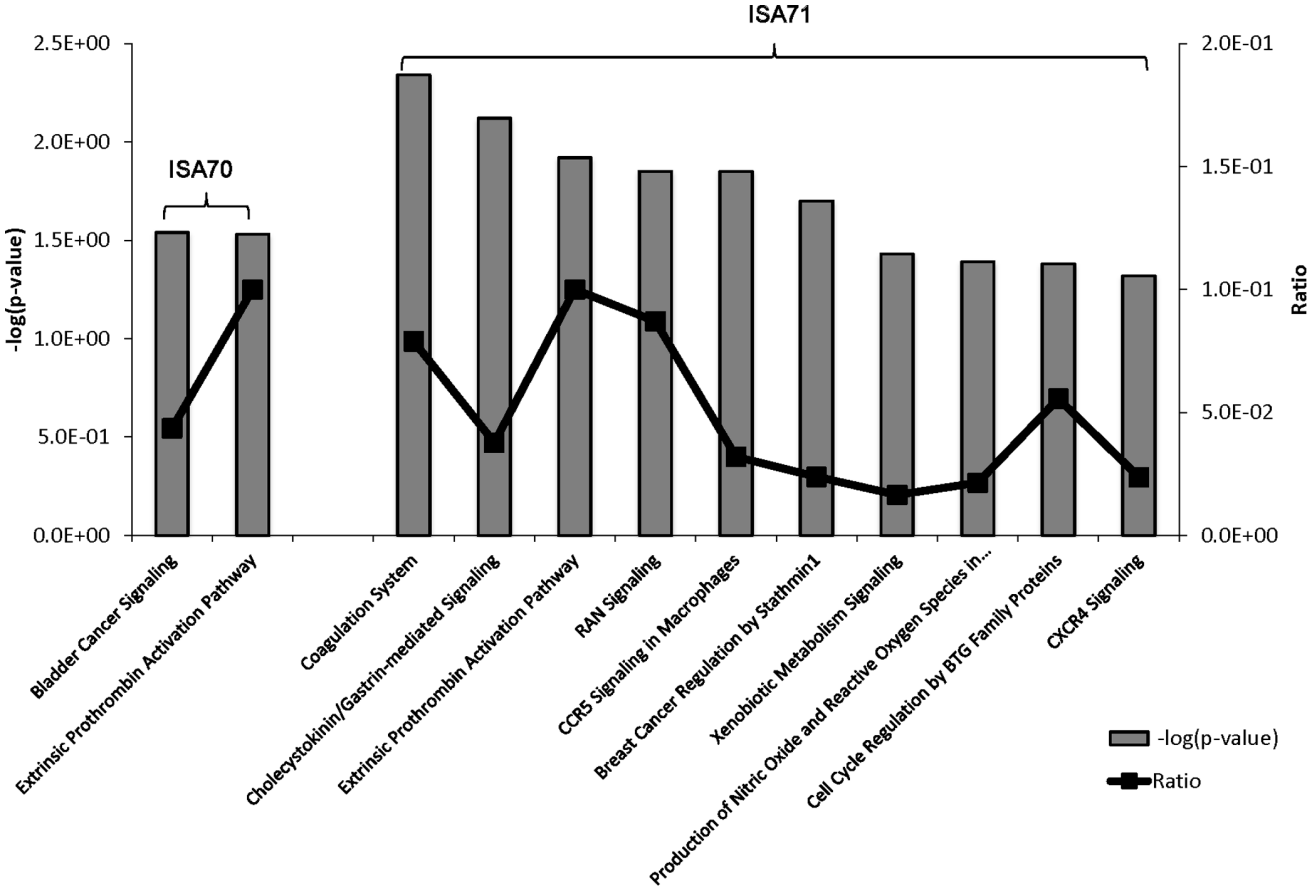
IPA canonic pathway analysis of differential transcript levels in chickens immunized with profilin plus ISA 70 *vs*. profilin alone and profilin plus ISA 71 *vs*. profilin alone. Illustrated are the pathways that were significantly associated with genes encoding the modulated transcripts in the comparisons of profilin plus ISA 70 *vs*. profilin alone (2 pathways) and profilin plus ISA 71 *vs*. profilin alone (10 pathways). The left ordinate and bars illustrates the statistical significance of each pathway expressed as the -log_10_ (*P* value) calculated using the right-tailed Fisher exact test. The right ordinate and line illustrate the ratio of the number of genes from the dataset that map to the indicated pathway divided by the total number of genes within that particular pathway.

The differently modified datasets were mapped to the corresponding genes of the human, mouse, and rat genomes using Ingenuity Knowledge Base software. From this analysis, 192 chicken genes were identified and annotated in the profilin/ISA 70 *vs*. profilin alone comparison, and 112 genes in the profilin/ISA 71 *vs*. profilin alone comparison. Biological function analysis using the IPA database identified the category “Disease and Disorder” as the most significantly associated with the genes identified in both comparisons, with 25 and 24 significantly associated biological functions respectively (Table 4). Of these, two biological functions were uniquely associated with the profilin/ISA 70 *vs*. profilin alone comparison, “Endocrine System Disorders” and “Nutritional Disease”, and one biofunction was exclusively associated with the profilin/ISA 71 *vs*. profilin alone comparison, “Antimicrobial Response”. Finally, the IPA database was used to identify the canonical pathways associated with the respective biofunctions of the two comparison groups. Two pathways were identified in the profilin/ISA 70 *vs*. profilin alone comparison, while 10 pathways were recognized in the profilin/ISA 71 *vs*. profilin alone comparison (Figure 2).

**Table 4:** Comparisons of biological functions in the category of “Diseases and Disorders” of the differentially expressed transcripts following vaccination with profilin plus ISA 70 *vs*. profilin alone and profilin plus ISA 71 *vs*. profilin alone.

Biological Function^1^	Profilin/ISA 70 *vs*. Profilin^2^	Biological Function^1^	Profilin/ISA 71 *vs*. Profilin^2^
Genetic Disorder	0.000114–0.0296	Genetic Disorder	0.000597–0.0283
Reproductive System Disease	0.000114–0.0229	Hematological Disease	0.000597–0.0283
Infectious Disease	0.000522–0.0239	Cancer	0.000704–0.0265
Inflammatory Response	0.000522–0.0305	Organismal Injury & Abnormalities	0.000880–0.0265
Hypersensitivity Response	0.00154–0.0229	Gastrointestinal Disease	0.00195–0.0187
Developmental Disorder	0.00168–0.0230	Hepatic System Disease	0.00195–0.0187
Cardiovascular Disease	0.00499–0.0229	Neurological Disease	0.00290–0.0283
Respiratory Disease	0.00499–0.0229	Inflammatory Response	0.00404–0.0283
Organismal Injury & Abnormalities	0.00649–0.0251	Connective Tissue Disorders	0.00457–0.0142
Immunological Disease	0.00737–0.0229	Inflammatory Disease	0.00457–0.0142
Ophthalmic Disease	0.00737–0.0229	Respiratory Disease	0.00457–0.0142
Hematological Disease	0.00737–0.0251	Renal and Urological Disease	0.00532–0.0142
Inflammatory Disease	0.00749–0.0229	Metabolic Disease	0.00532–0.0283
Endocrine System Disorders	0.00761–0.0229	Dermatological Diseases & Condition	0.00580–0.0283
Metabolic Disease	0.00761–0.0229	Cardiovascular Disease	0.00677–0.0142
Gastrointestinal Disease	0.00761–0.0296	Immunological Disease	0.00677–0.0283
Cancer	0.0114–0.0296	Infectious Disease	0.00838–0.0265
Dermatological Diseases & Condition	0.0169–0.0229	Auditory Disease	0.0142–0.0283
Hepatic System Disease	0.0169–0.0229	Developmental Disorder	0.0142–0.0283
Renal and Urological Disease	0.0194–0.0251	Ophthalmic Disease	0.0142–0.0283
Auditory Disease	0.0229	Hypersensitivity Response	0.0102
Connective Tissue Disorders	0.0229	Antimicrobial Response	0.0142
Neurological Disease	0.0229	Reproductive System Disease	0.0142
Nutritional Disease	0.0229	Skeletal & Muscular Disorders	0.0142
Skeletal & Muscular Disorders	0.0229		

1 Datasets were analyzed by BioFunction analysis using IPA software.

2 *P* values were calculated using the right-tailed Fisher exact test and are listed in decreasing order of statistical significance.

### Effect of Vaccination with Profilin Plus ISA 71 on Body Weight Gain and Oocyst Shedding

**Figure 3:**
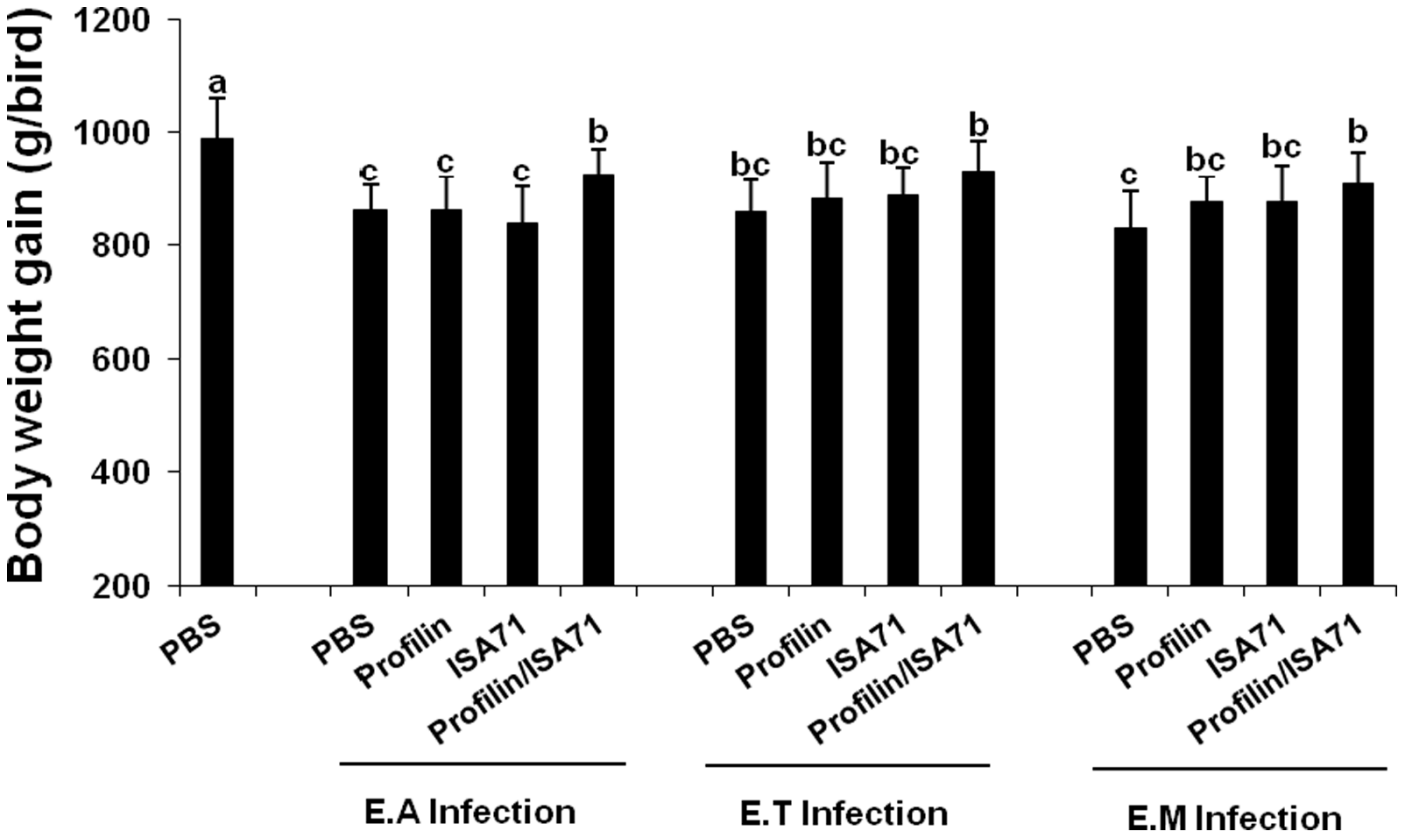
Effect of vaccination with profilin plus ISA 71 on body weight gain following infection with *E. acervulina, E. tenella*, or *E. maxima*. Chickens were subcutaneously vaccinated with PBS, profilin alone, ISA 71 alone, or profilin plus ISA 71 at 7 and 14 days post-hatch. At 7 days post-secondary vaccination, the chickens were uninfected or infected with 1.0×10^4^ sporulated oocysts of *E. acervulina* (E.A), *E. tenella* (E.T), or *E. maxima* (E.M). Body weight gains were measured between 0 and 10 days post-infection. Each bar represents the mean ± S.D. value (n = 8). Within each graph, bars with different letters are significantly different according to the Duncan’s multiple range test (*P*<0.05).

**Figure 4:**
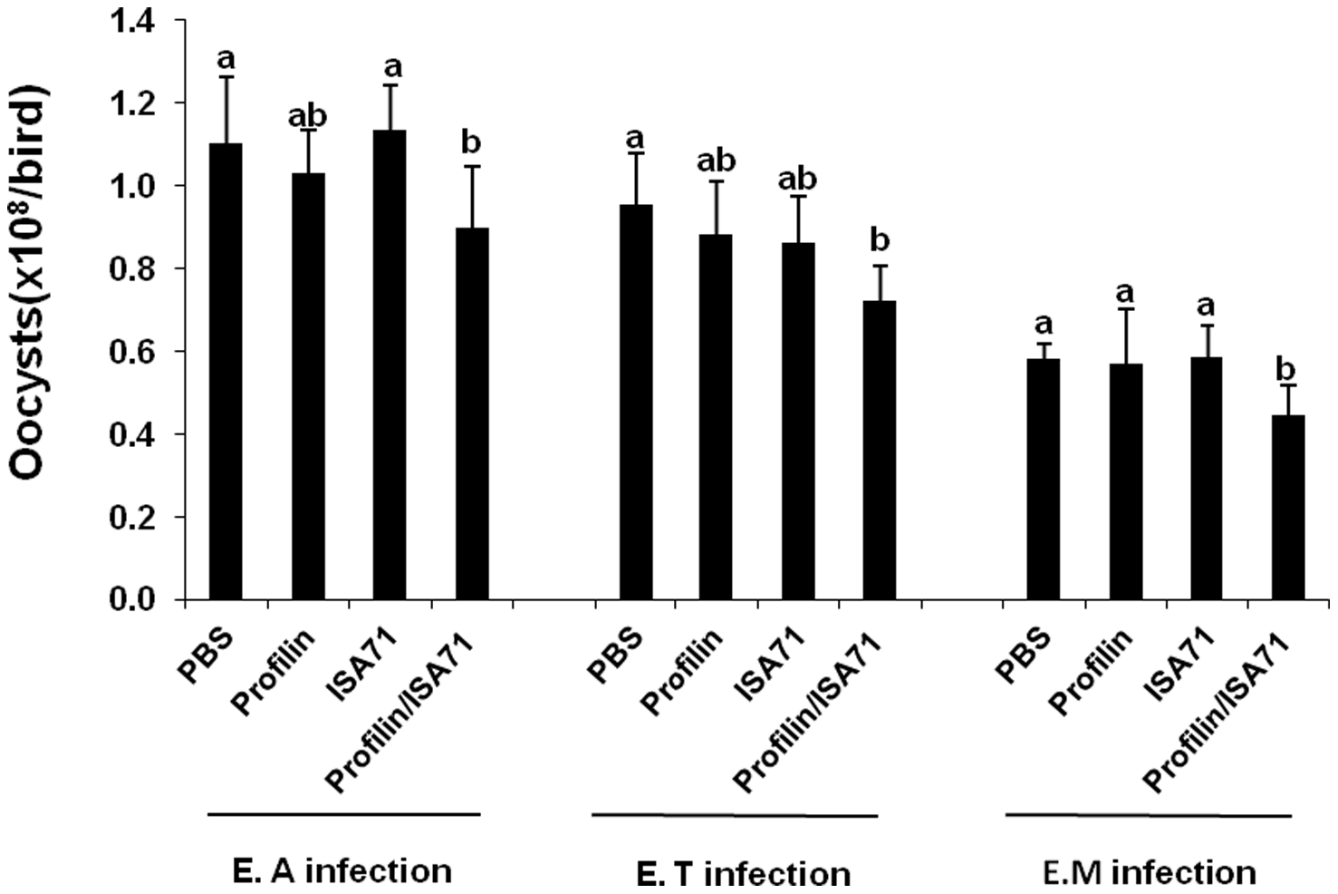
Effect of vaccination with profilin plus ISA 71 on fecal oocyst shedding following infection with *E. acervulina, E. tenella*, or *E. maxima*. Chickens were vaccinated and infected as described in Figure 3. Fecal oocyst numbers were measured between 6 and 10 days post-infection. Each bar represents the mean ± S.D. value (n = 8). Within each graph, bars with different letters are significantly different according to the Duncan’s multiple range test (*P*<0.05).

**Figure 5:**
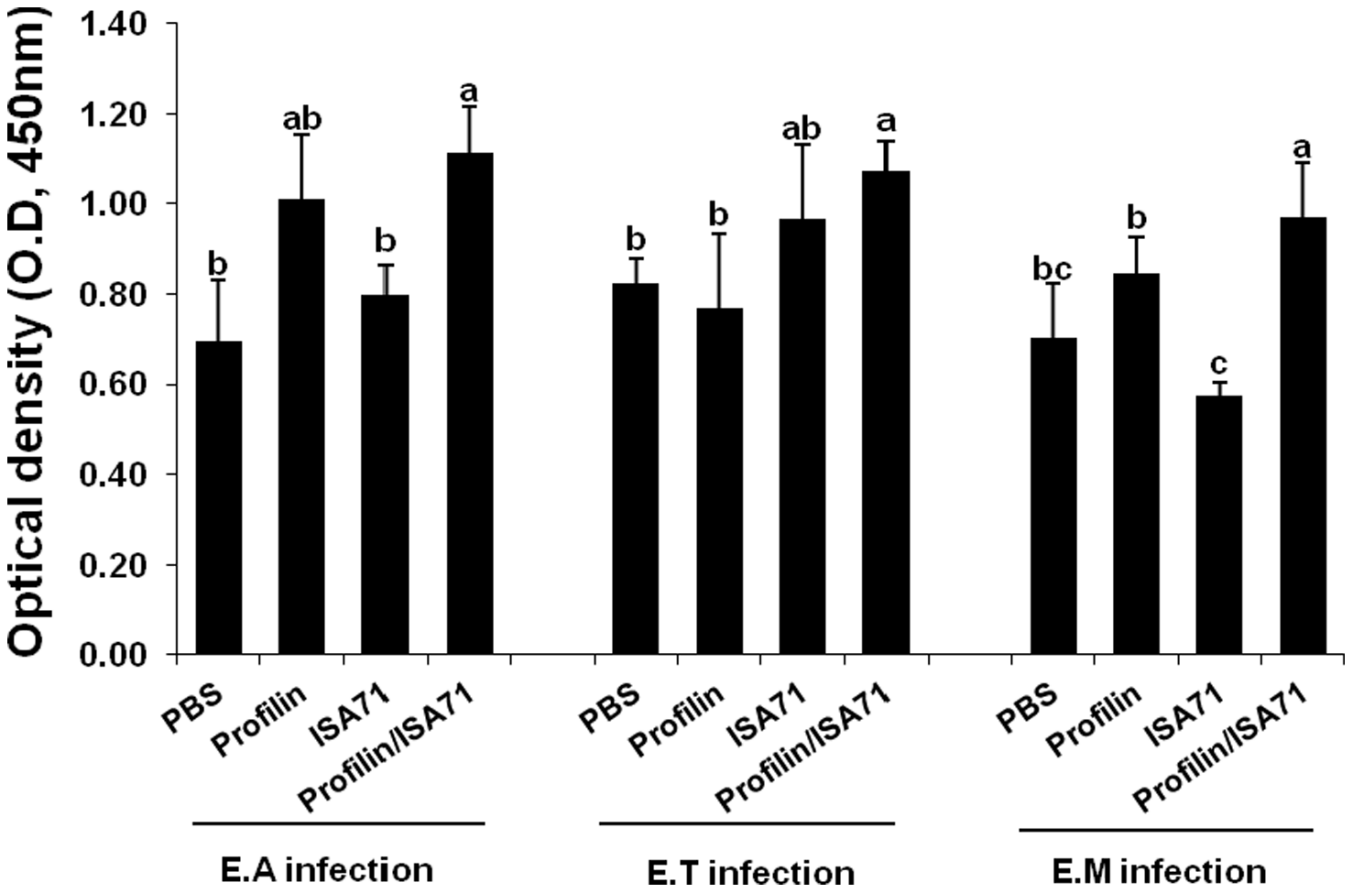
Effect of vaccination with profilin plus ISA 71 on serum anti-profilin antibody levels following infection with *E. acervulina, E. tenella*, or *E. maxima*. Chickens were vaccinated and infected as described in Figure 3. Serum anti-profilin antibody levels were measured by ELISA at 3 days post-infection. Each bar represents the mean ± S.D. value (n = 5). Within each graph, bars with different letters are significantly different according to the Duncan’s multiple range test (*P*<0.05).

Because immunization with profilin plus ISA 71 *vs*. profilin alone was associated with more unique biological functions, as well as a larger number of total genes and pathways associated with these functions, compared with profilin/ISA 70 *vs*. profilin alone, we focused on ISA 71 as a possible adjuvant for *in vivo* protection studies against experimental *Eimeria* infection following profilin vaccination. Chickens vaccinated with profilin plus ISA 71 and infected with *E. acervulina* had increased body weight gains between 0 and 10 days post-infection compared with infected chickens vaccinated with profilin alone (Figure 3). Profilin/ISA 71-vaccinated and *E. maxima*-infected animals had decreased oocyst fecal shedding between days 6 and 10 post-infection compared with infected chickens vaccinated with profilin alone (Figure 4). Finally, profilin/ISA 71-vaccinated birds that were infected with *E. tenella*- or *E. maxima* had greater anti-profilin serum antibody levels at 3 days post-infection compared with infected birds vaccinated with profilin alone (Figure 5).

### Effects of Vaccination with Profilin Plus ISA 71 Adjuvant on Cytokine Transcript Levels in Intestinal IELs

**Figure 6:**
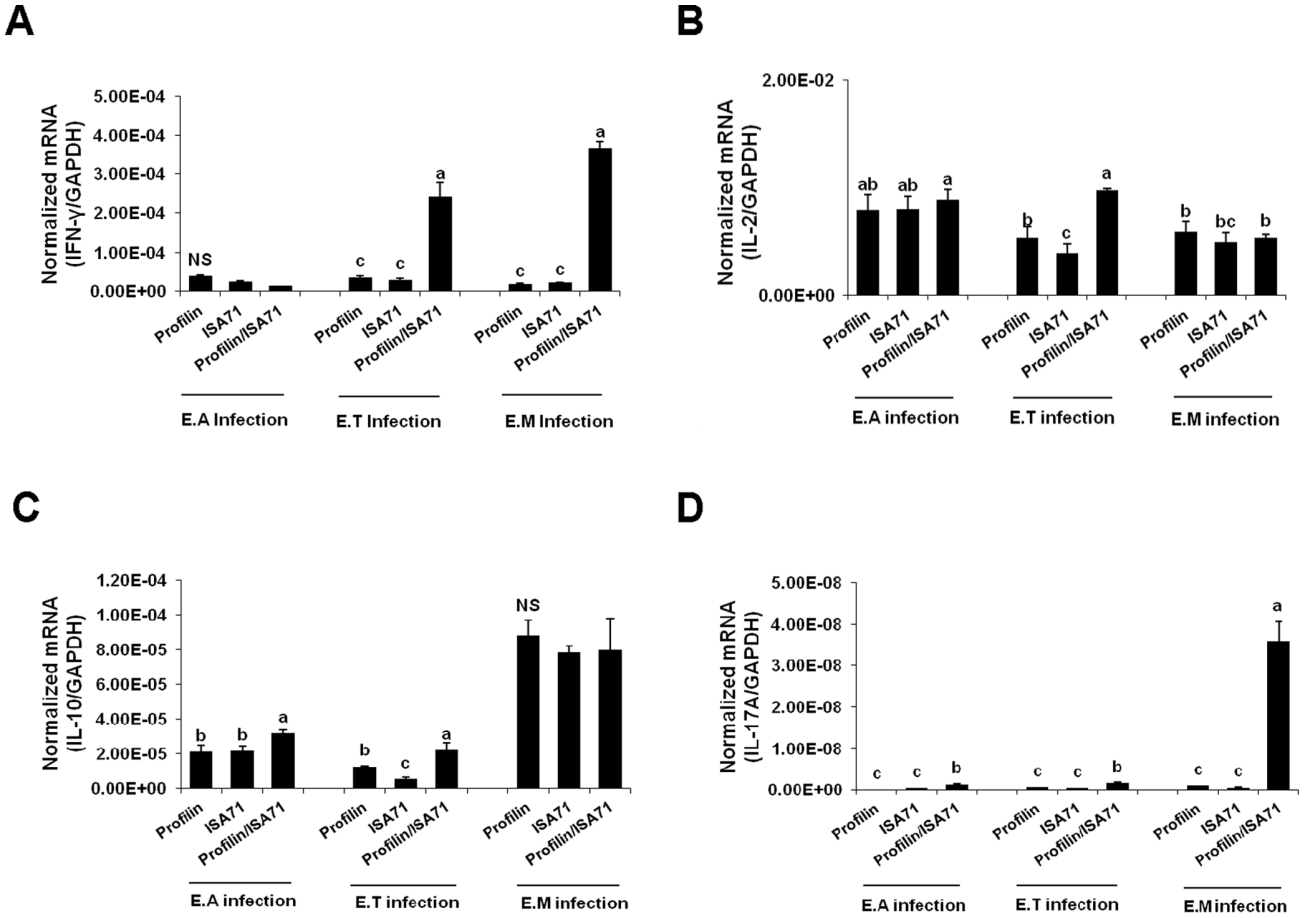
Effect of vaccination with profilin plus ISA 71 on intestinal IEL cytokine transcript levels following infection with *E. acervulina, E. tenella*, or *E. maxima*. Chickens were vaccinated and infected as described in Figure 3. Intestinal IEL transcripts for IFN-γ (A), IL-2 (B), IL-10 (C), and IL-17A (D) were measured by quantitative RT-PCR at 3 days post-infection and normalized to GAPDH transcript levels. Each bar represents the mean ± S.D. value (n = 3). Within each graph, bars with different letters are significantly different according to the Duncan’s multiple range test (*P*<0.05). NS, not significant.

Chickens vaccinated with profilin plus ISA 71 and infected with all 3 *Eimeria* parasites had greater levels of intestinal IEL gene transcripts encoding IFN-γ, IL-2, IL-10, and/or IL-17A at 3 days post-infection compared with infected birds vaccinated with profilin alone (Figure 6). The greatest cytokine transcriptional response was seen in the *E. tenella*-infected group. More specifically, *E. tenella*-infected animals had increased levels of all 4 transcripts, while *E. acervulina*-infected birds had greater IL-10 and IL-17A mRNA levels and *E. maxima*-infected birds had increased levels of IFN-γ and IL-17A mRNAs.

## Discussion

This study demonstrated that chickens immunized with profilin plus ISA 70 or profilin plus ISA 71 exhibited spleen lymphocyte transcriptome changes, compared with immunization with profilin alone, that were consistent with alteration of levels of mRNAs encoded by genes belonging to the IPA database category “Disease and Disorder”. Compared with profilin/ISA 70 *vs*. profilin alone, the profilin/ISA 71 *vs*. profilin alone comparison was associated with more unique biological functions, and a larger number of genes and pathways associated with these functions, suggesting that vaccination with profilin/ISA 71 may induce a greater protective host response to experimental *Eimeria* infection. Compared with profilin alone, vaccination with profilin/ISA 71 was correlated with (a) increased body weight gains following *E. acervulina* infection, (b) reduced parasite fecal shedding following *E. maxima* infection, (c) augmented anti-profilin serum antibody titers in *E. maxima*- and *E. tenella*-infected chickens, and (d) higher IFN-γ, IL-2, IL-10, and IL-17A transcript levels in gut IELs of *E. acervulina*-, *E. maxima*-, and/or *E. tenella*-infected chickens. Weight gains and parasite shedding were equal in the ISA 71 alone *vs*. PBS negative control groups, and IEL transcript levels were generally equivalent in the ISA 71 only *vs*. profilin alone groups, indicating that the adjuvant itself was not responsible for these effects. Collectively, these data suggest that vaccination of chickens with profilin plus ISA 71 may increase resistance against experimental avian coccidiosis by selective *Eimeria* species.

In general, subunit vaccines against many infectious diseases of livestock and poultry in the absence of adjuvants are weakly immunogenic, and repeated vaccinations are often needed to generate sufficient protective immunity to control infection [22]. While immunologic adjuvants are known to stimulate the host's immune system response to a target antigen, without themselves conferring immunity, the limited availability of safe and efficacious adjuvants for veterinary use hampers disease control strategies against some of the more common infectious pathogens of food animals. The Montanide ISA series of adjuvants are ready-to-use W/O, O/W or W/O/W emulsions, incorporating high-grade, injectable mineral or non-mineral oils. Proven benefits in veterinary medicine include the production of stable vaccine emulsions with low viscosity, ease of injection, reduced toxicity, and induction of a strong, long-lasting immune response [6], [7], [23], [24], [25], [26], [27]. Montanide ISA adjuvants appear to be ideally suited for use with vaccines of limited immunogenicity, such as profilin, an intracellular component that contributes to actin-dependent gliding motility and host cell invasion of apicomplexan parasites, including *Toxoplasma gondii* and *Eimeria* spp. [28]. *T. gondii* profilin binds to Toll-like receptor-11, inducing a potent IL-12 response in dendritic cells, and resulting in enhanced humoral and Th1 cellular immune responses against the parasite [28], [29]. The *E. acervulina* protein, 3-1E, was identified earlier in merozoites of the microorganism as an immunogenic component of the parasite which induced high levels of antigen-specific proliferation and IFN-γ production by chicken splenic lymphocytes [30]. Polyclonal antibodies raised against *E. acervulina* 3-1E cross-reacted with the homologous proteins of *E. tenella* and *E. maxima*. Subsequently, 3-1E was shown to be the *Eimeria* homologue of *T. gondii* profilin [28].

We previously reported that compared with unimmunized controls, chickens immunized with profilin in the absence of adjuvant had altered levels of 127 gene transcripts (71 up-regulated, 56 down-regulated). The total number of transcripts affected by profilin/ISA 70 *vs*. profilin alone (509) or profilin/ISA 71 *vs*. profilin alone (296) observed in the current investigation is comparable to the results of our previous studies using other adjuvants and immunomodulators. More specifically, compared with chickens immunized with profilin alone, chickens given profilin plus the Quil A/cholesterol/dimethyl dioctadecyl ammonium bromide/Carbopol (QCDC) adjuvant mixture had 164 altered mRNAs (60 up-regulated, 104 down-regulated), and birds immunized with profilin plus QCDC incorporating the Bay R1005 immunostimulant (QCDCR) had 233 modulated transcripts (103 up-regulated, 130 down-regulated) [31]. In a subsequent study in the absence of profilin vaccination, dietary supplementation of chickens with propyl thiosulfinate, a secondary metabolite of garlic with immunoenhancing properties, identified 1,227 transcripts whose levels were altered in intestinal IELs compared with untreated controls [13]. As in the current report, biological pathway analysis identified the propyl thiosulfinate-altered transcripts to be encoded by genes associated with the IPA category “Disease and Disorder”.

Interestingly, whereas ISA 70 and ISA 71 are composed of similar adjuvant formulations, only 22 common transcripts were shared between the profilin/ISA 70 *vs*. profilin alone and the profilin/ISA 71 *vs*. profilin alone groups, representing 4.3% and 7.4% of the total number of altered mRNAs, respectively. On the other hand, comparison of profilin/ISA 70-immunized chickens with the profilin/ISA 71 group identified 315 altered transcripts, indicating that the number of dissimilar mRNAs was substantially greater than the number of shared transcripts. Correspondingly, a similar comparison using the QCDC and QCDCR adjuvants revealed 397 altered transcripts in the profilin/QCDC *vs*. profilin/QCDCR groups [31]. In another study, comparative microarray analysis between uninfected *vs*. *E. acervulina*, *E. tenella*, or *E. maxima* infections was used to identify commonly altered transcripts in these 3 denoted groups [12]. Following *E. acervulina* infection, 2,431 mRNAs were altered, while infection with *E. tenella* and *E. maxima* modulated the levels of 2,522 and 1,717 mRNAs respectively. From these, 766 transcripts were common to *E. acervulina* and *E. tenella*, 319 were shared between *E. acervulina* and *E. maxima*, 289 were common to *E. tenella* and *E. maxima*, and 361 mRNAs were shared between all 3 infections. Taken together, these results indicate that infection with intact, viable coccidia parasites stimulates a greater host transcriptional response compared with profilin vaccination in the presence or absence of adjuvant.

Body weight gain and fecal oocyst shedding are reliable clinical signs for the evaluation of protective immunity in avian coccidiosis [32]. Both parameters are directly correlated with the levels of intestinal proinflammatory cytokines in *Eimeria*-infected chickens [33], [34], [35]. IFN-γ plays a critical role in the *Eimeria*-stimulated host immune response, and is one of the earliest cytokines detected in infected intestinal mucosa [36]. Indeed, IFN-γ is the dominant cytokine elicited in the gut of *Eimeria*-infected chickens that typifies the Th1 cell-mediated immune response seen during experimental avian coccidiosis [37]. Endogenous IFN-γ production in gut epithelia was positively associated with improved body weight gain and decreased oocyst shedding in birds following *Eimeria* infection [33]. Chickens treated exogenously with puified recombinant IFN-γ protein showed greater weight gain and reduced fecal oocyst numbers following *E. acervulina* infection [38]. IL-2 and IL-17A are other members of this Th1 cytokine response that serve to recruit, activate, and amplify immune effector leukocytes with cytotoxic activtiy against coccidia parasites [39], [40]. On the other hand, IL-10 driven inhibition of IFN-γ production suggests that this counter-regulatory mediator may favor a shift toward a Th2 response later in the course of infection, and prevent tissue damage as a consequence of uncontrolled intestinal inflammation [41]. Therefore, augmented production of IFN-γ, IL-2, IL-17A, and IL-10 by profilin plus ISA 71 appears to preserve the natural balance of pro- and anti-inflammatory pathways in the gut that are necessary for an effective cellular immune response against the invading parasite while maintaining tissue homeostasis.

In conclusion, this study identified transcriptome dynamics in chickens following immunization with the *Eimeria* recombinant profilin protein in combination with either the ISA 70 or ISA 71 W/O adjuvants by comparison with immunization with profilin alone. Based on the greater transcriptional response elicited by profilin/ISA 71, this antigen/adjuvant mixture was used to subsequently demonstrate increased protection against experimental avian coccidiosis, as assessed by augmented body weight gains, decreased parasite fecal shedding, greater anti-profilin serum antibody titers, and increased levels of cytokine gene transcripts compared with vaccination with profilin alone. These results suggest that profilin in conjunction with ISA 71 provides an effective means of eliciting humoral and cellular immune responses with the potential to generate protective immunity against *Eimeria* infection.
